# Ultrasonic characteristics and equivalent crack width of coal and rock bodies around boreholes during progressive failure

**DOI:** 10.1371/journal.pone.0285808

**Published:** 2023-05-25

**Authors:** Xiang Ji, Tianjun Zhang, Lei Zhang, Wen Yang, Hang Zhang

**Affiliations:** 1 College of Safety Science and Engineering, Xi’an University of Science and Technology, Xi’an, China; 2 Key Laboratory of Western Mine Exploitation and Hazard Prevention of the Ministry of Education, Xi’an, China; 3 College of Energy, Xi’an University of Science and Technology, Xi’an, China; Al Mansour University College-Baghdad-Iraq, IRAQ

## Abstract

The ultrasonic characteristics of the coal and rock bodies around boreholes during failure are closely related to the crack propagation law. To investigate the ultrasonic characteristics and crack propagation law of coal and rock bodies around boreholes, different grouting samples with boreholes were taken to carry out ultrasonic test during progressive failure. The ultrasonic amplitude, velocity and attenuation coefficient of the samples were analyzed. According to the ultrasonic time difference formula, the equivalent crack width of the sample during the failure process is calculated. The influence of grouting material on the crack propagation law is quantitatively analyzed. The results show that: (1) The peak stress, elastic energy at the peak, ultrasonic parameters and crack propagation of the coal and rock bodies around boreholes show obvious differences influenced by the strength of the grouting material. (2) During the loading process, the arrival time of the first wave of the sample with holes is 5μs later than that of the grouting sample, and the ultrasonic energy attenuates fastest in the time domain, and the coda wave is not developed. (3) During the progressive failure, the ultrasonic velocity and attenuation coefficient of all show three stages of stability(0~0.6*σ*_p_), slow change(0.6*σ*_p_~0.8*σ*_p_) and rapid change(0.8*σ*_p_~1.0*σ*_p_). According to the "sudden decrease" of velocity and the "sudden increase" of attenuation coefficient to judge the crack propagation of sample. (4) The equivalent crack width of the sample increases exponentially with the increase of stress level. At the time of reaching the peak stress, the equivalent crack width of SH-BH increases about 0.027mm~0.032mm, SH-PU about 0.01mm~0.014mm, and SH-CEM about 0.002mm~0.006mm.

## Introduction

Efficient extraction of gas energy from mines not only reduces gas disasters, but also plays an important role in improving China’s energy structure and helps to achieve the goal of "carbon neutrality" [1,2]. In recent years, China’s gas extraction and utilization technology has made progress, but there is still unbalanced extraction, and the overall low extraction concentration and extraction rate [3,4]. The reason is not only the poor permeability of coal seam [5], but also that under the influence of some factors such as permeability enhancement, tunneling and mining. The special structure of the borehole leads to the failure of coal around the borehole [6], resulting in a large number of crack networks, forming air leakage channels and seriously reducing the concentration of gas extraction [7]. To improve the effect of gas extraction, it is necessary to analyze the crack propagation mechanism of the coal and rock bodies around boreholes and explore the development of the crack channel around boreholes using specific technical measures [8,9].

In the past few years, most scholars have used various laboratory techniques to detect the deformation failure process of coal and rock bodies and analyze the crack propagation law. These detection techniques include digital scatter correlation measurement (DSCM) [10], scanning electron microscopy (SEM) [11], computed tomography (CT) [12] and acoustic emission (AE) [13]. However, the strict testing conditions of the above technology make it difficult to be applied in field engineering [14]. In recent years, ultrasonic testing has played an important role in the field of coal and rock mechanics as a new technology that is active non-destructive, rapid and simple [15]. Due to the strong sensitivity of ultrasound to cracks, it can reflect the internal crack propagation in coal and rock bodies more accurately, so many scholars have carried out a lot of basic research on the correlation between ultrasonic and stress, ultrasonic response of crack propagation [16]. Mohamed et al. [17], Wang et al. [18] and Wang et al. [19] considered that the ultrasonic characteristic parameters, such as ultrasonic waveform, velocity, and attenuation coefficient, are well correlated with the physical and mechanical properties of coal and rock bodies, which can reflect the failure process of coal and rock bodies more accurately.

For the study of ultrasonic velocity, Hou et al. [20] established a model for calculating four rock mechanical parameters based on the crack complexity and ultrasonic velocity of coal seams, and analyzed the relationship between mechanical parameters and ultrasonic velocity of coal and rock bodies. Yang et al. [21] quantitatively analyzed the influence of joint thickness and water volume content on ultrasonic velocity. Zhang et al. [22], Gondim et al. [23] used ultrasonic transmission velocities (UTV) measurement device to study change characteristics of ultrasonic velocity of the coal and rock bodies during the failure process, then evaluated the stability of coal and rock bodies. Jia et al. [24] carried out the ultrasonic velocity test of coal and rock bodies under loading, and analyzed the crack propagation process of coal and rock bodies. Wang et al. [25] established a fractal ultrasonic velocity evolution model of water-bearing coal based on fractal theory, and studied the relationship between crack structure parameters and ultrasonic velocity. In the study of ultrasonic waveform and attenuation coefficient, Pahlavan et al. [26] studied the effect of crack width on ultrasonic transmission time and amplitude during unloading of concrete structures. Xie et al. [27] proposed a field quantitative test method for rock elastic modulus based on ultrasonic testing, and discussed the mechanical properties of rock. Gheibi et al. [28] used ultrasonic technology to monitor the failure law of sandstone rock in the shear process and analyzed the propagation characteristics of ultrasonic waves through rock joints. Zhang et al. [29] realized the quantitative prediction of the evolution process of crack rock bodies through attenuation coefficient. Based on the assumption of the ultrasonic exponential decay [30] and the frequency-independent *Q* model, Matsushima et al. [31] explored the ultrasonic attenuation mechanism in porous rocks. Guo et al. [32] carried out uniaxial compression tests of different heat treatment samples, and used a combination of active ultrasound and passive acoustic emission to monitor the failure characteristics of the samples.

The above research shows that ultrasonic wave propagation in fractured coal and rock bodies is a typical elastic wave propagation problem. From the aspects of ultrasonic velocity, waveform and attenuation coefficient, the transmission law of ultrasonic in coal and rock bodies is explored by theoretical analysis, experimental research and numerical calculation. However, by the large, the following deficiencies remain: (1) The propagation process and characteristics of ultrasonic wave in the coal and rock bodies around boreholes are still undefined. (2) At present, most studies tend to analyze ultrasonic parameters, such as ultrasonic amplitude, ultrasonic velocity and dominant frequency, to evaluate the failure of coal and rock bodies, and lack quantitative analysis of crack expansion law in the failure process of coal and rock bodies. In this paper, the transmission characteristics of ultrasonic in the coal and rock bodies around boreholes during failure are analyzed. Various parameters of ultrasonic are collected to investigate the relationship between ultrasonic transmission and the stress of the coal and rock bodies around boreholes. In addition, based on the ultrasonic time difference formula, the calculation method of equivalent crack width is given to quantitatively study the crack propagation law of coal and rock bodies around boreholes, which can provide the theoretical basis for crack detection engineering of coal bodies around the gas extraction borehole.

## Theory

The ultrasonic detector used in this experiment includes transmitter and receiver of ultrasonic. According to international standard ISO1920-7:2004 [33], the transmitter and receiver of ultrasonic should be installed on two parallel surfaces of the sample with a certain length (*D*), and trigger a series of ultrasonic pulses. By analyzing the ultrasonic amplitude, velocity and attenuation coefficient, the ultrasonic response characteristics of internal cracks in coal and rock bodies during loading were studied. In the following we will describe in detail the determination methods of these three ultrasonic parameters.

**Fig 1:**
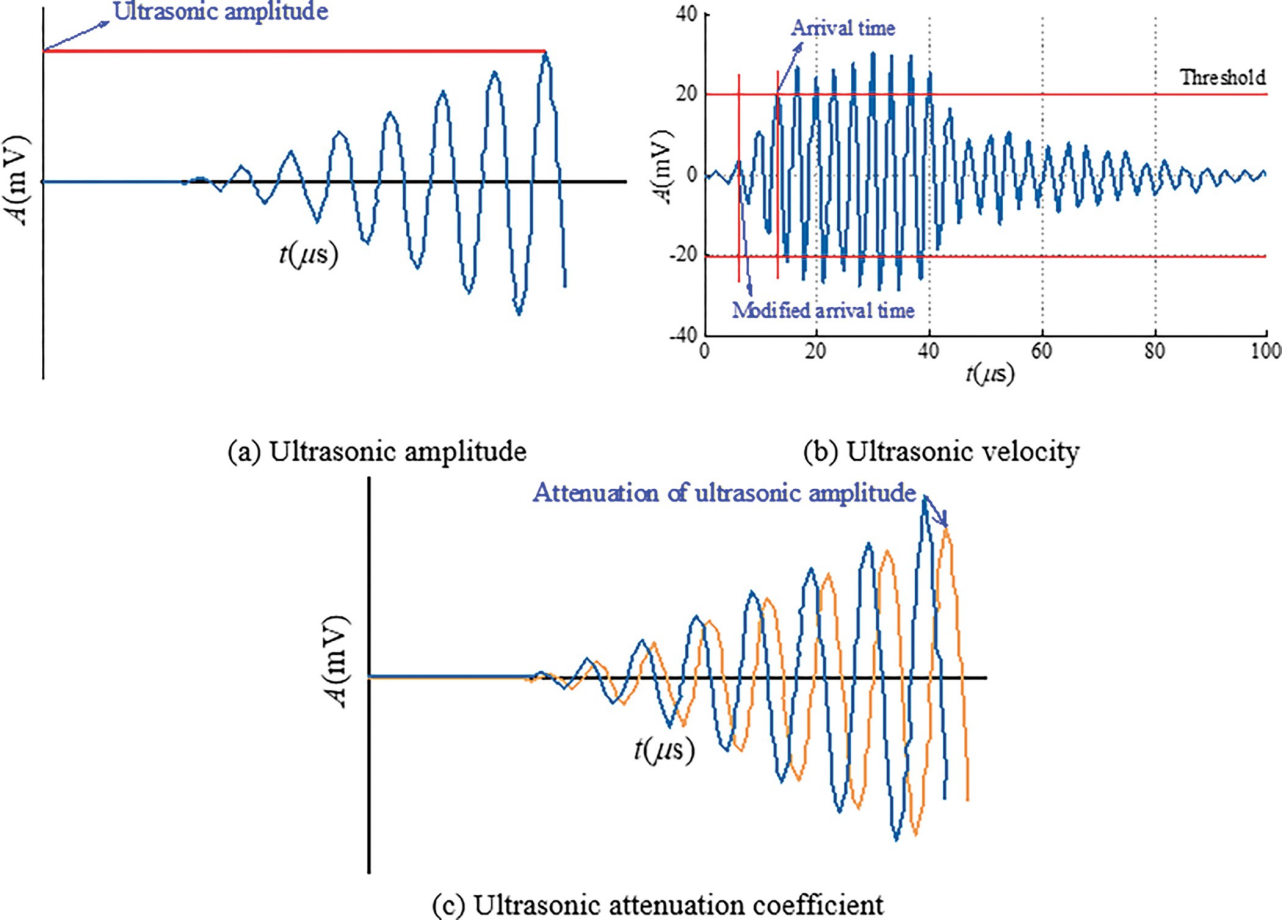
The determination of ultrasonic parameters (modified from [34]).

In the experiment, the waveform data, the ultrasonic emission time *t*_0_ and the ultrasonic receiving time *t*_1_ of the sample during loading are tested and recorded, from which the ultrasonic amplitude, velocity and attenuation coefficient can be obtained. The ultrasonic amplitude is the maximum value of the waveform data, and its determined value is shown in Fig 1(A). Because the ultrasonic wavelength is much smaller than the sample size, the coal and rock bodies sample can be regarded as an infinite medium, and the calculation of ultrasonic velocity is shown in Fig 1(B). Using the signal comparison test method, the ultrasonic attenuation coefficient is calculated according to the first ultrasonic amplitude before and during the loading, and the distance between the ultrasonic transmitter and the ultrasonic receiver, as shown in Fig 1(C).

Before calculating the ultrasonic velocity, it is necessary to calibrate the arrival time of the received ultrasonic signal. Then according to the ultrasonic time difference formula, the ultrasonic velocity of the sample during loading is calculated [35]:

$$V_{p}=D/\Delta t$$ (1)

Where *V*_p_ is the test of ultrasonic velocity, m/s; *D* is the distance of ultrasonic propagation in the sample, which is the length of the sample, m; *Δt* is time difference, *Δt* = *t*_1_-*t*_0_, s。

The equation of ultrasonic attenuation coefficient is [36]:

$$\alpha =(\ln A_{m}-\ln A_{i})/D$$ (2)

Where *α* is the ultrasonic attenuation coefficient; *A*_m_ is the first ultrasonic amplitude before loading, mV; *A*_i_ is the first ultrasonic amplitude during loading, mV.

## Materials and methods

### Sample preparation

The chemical composition, structure and physical and mechanical properties of coal indicate that coal samples are prone to structural defects and brittle fractures during the preparation process [37,38]. According to the results of References [39,40], the mechanical properties of gypsum samples (such as compressive strength, elastic modulus, Poisson’s ratio, etc.) are close to the mechanical properties of coal and rock. The crack law of gypsum sample and raw coal sample is basically the same during progressive failure. According to the final failure image of the sample, it can be seen that the macroscopic main cracks of the sample are diagonally damaged on the left and right sides of the borehole [41–45].

**Fig 2:**
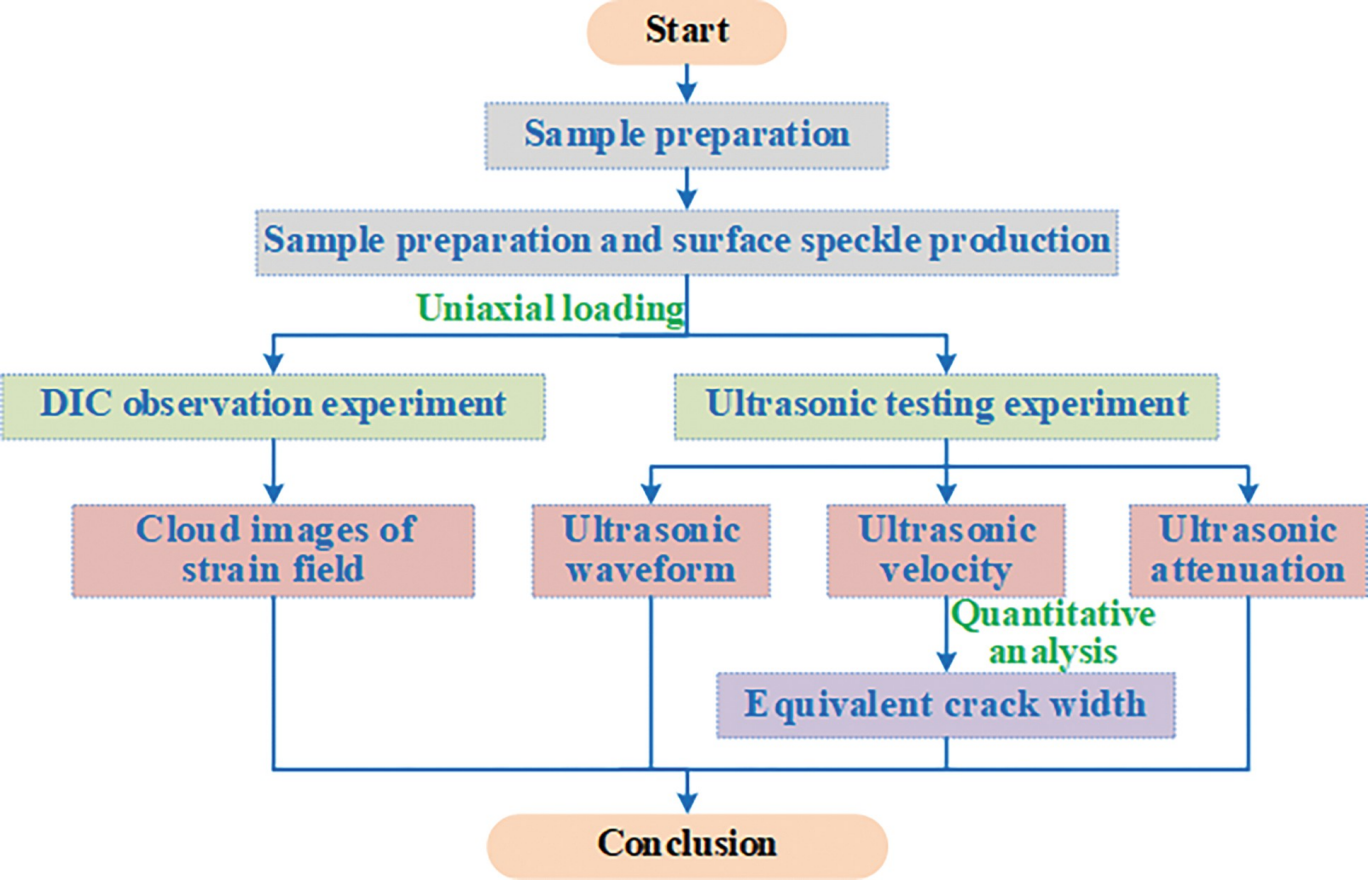
Test flowchart.

To observe the cracks produced by the brittle fracture of a sample and avoid plastic deformation, this paper is based on a similar simulation test study, the slurry was made by mixing gypsum and water with a mass ratio of 7:3, and was filled in a 70 mm×70 mm×140 mm rectangular sample mold, and was prefabricated with *φ* = 10 mm cylindrical boreholes in the center to simulate gas drainage boreholes (as shown in Fig 2). After the samples were first set into shape, and all samples will be divided into 3 groups of 5 samples each. Then, the grouting materials commonly used in coal mines were filled in the boreholes: polyurethane and expansion cement [46,47]. All samples were placed in a concrete standard curing box for 28 days, and then the surfaces of the samples were polished and repaired to ensure the dimensional accuracy and surface flatness of the samples. Finally, the samples are named according to "sample type—grouting material—number", for example: SH-BH-1 represents 1# sample with boreholes; SH-PU-2 represents 2# polyurethane injection sample; SH-CEM-3 represents 3# expansion cement injection sample.

### Test process

In order to ensure the homogeneity of the specimens and reduce the test error caused by the sample preparation process, all specimens were tested by RSM-SY7 ultrasonic detector before the test started. In each group, three samples with stable waveforms and consistent initial wave velocity were selected for progressive failure test [48,49]. After the sample selection is completed, the speckle field on the surface of the sample is made by spraying paint to improve the accuracy of the digital speckle test [50]. The specific production steps of speckle field are as follows: white paint was sprayed on the test surface of the specimen, and after it dried, black paint was sprayed to create a random speckle pattern, which can be repeated several times to ensure that the spraying process is as uniform as possible. Finally, the progressive failure test was carried out by using the ultrasonic test system of coal and rock deformation and failure. The specific test process is shown in Fig 2.

**Fig 3:**
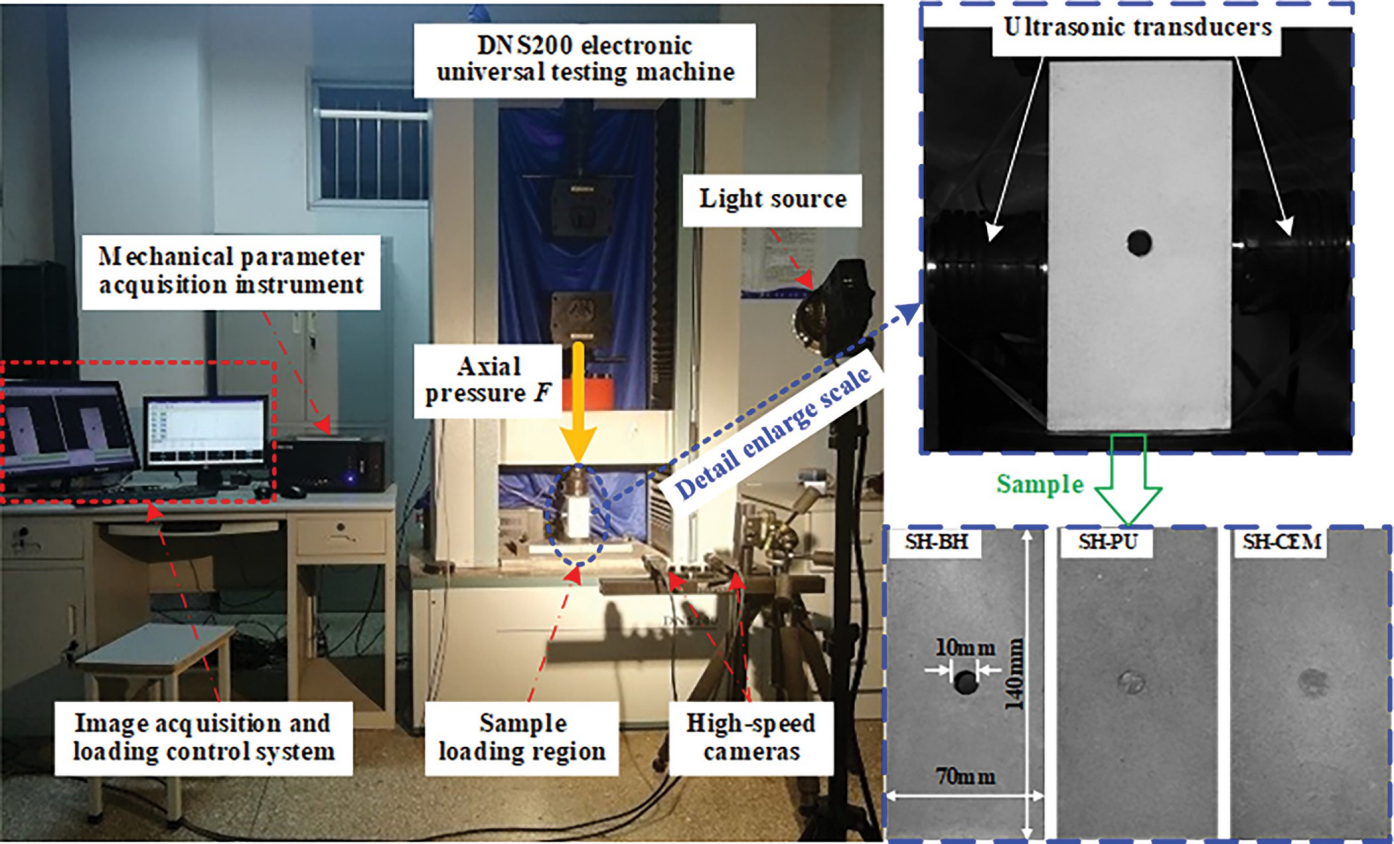
Test system and sample.

The test system mainly included the stress loading system, the digital image correlation system (DIC) and the ultrasonic testing system. The system arrangement is shown in Fig 3.

The stress loading system included a DNS200 electronic universal testing machine and a mechanical parameter acquisition instrument, and the testing machine supports two loading modes of load and displacement. During the test, the sample should be fully contacted with the indenter of the press to reduce the influence of the flatness on the test results. The displacement-controlled uniaxial loading method was used in the test. The loading rate was 0.05 mm/min, and the data acquisition frequency of the press was 1 Hz. The test data can be collected in real time and the mechanical parameters can be recorded.

The DIC system used two sets of Point grey^®^ CCD cameras (GS3-PGE-50S5M-C, Point Grey Research, Ltd., Vancouver, Canada; sensor: Sony ICX625 CCD, 2/3"; resolution: 2448pixel×2048pixel; pixel size: 3.45μm; frame rate: 15fps), with Pentax® 75mm fixed-focus lenses (FA645 75mm f/2.8, Ricoh Imaging Company, Ltd., Tokyo, Japan; focal length: 75mm), and supporting software VIC-Snap^TM^ software to acquire test images in real time. The acquisition frequency of the image acquisition system in the experiment was 1 Hz. In order to prevent the influence of strobe on data acquisition, two Osram^®^ 55460 optical instrument lamps were used to fill in the light to obtain clear images of the strain field evolution process on the surface of the coal and rock bodies around boreholes. The ultrasonic testing system is a non-metallic ultrasonic tester RSM-SY7 (RSM-SY7, Mechanics Institute of Chinese Academy of Sciences, Hubei, China), which can collect ultrasonic time domain parameters and primary waveform in real time. The ultrasonic tester was set to continuous emission, and the ultrasonic acquisition length was set to 512μs. Before the test begins, a standard aluminum block sample of *φ*50mm×100mm is used to calibrate the ultrasonic tester [51]. Before the experiment, the first ultrasonic measurement was performed to obtain the initial ultrasonic waveform of the sample, and the initial ultrasonic parameters of the sample were calculated. Subsequently, waveform data was acquired every 1kN during the loading process until the sample was completely damaged.

### Test results

The mechanical and ultrasonic testing results of coal and rock bodies around boreholes are shown in Table 1. Where the elastic energy at the peak stress *U*_e_ is calculated by formula $U_{e}=Ah\sigma_{p}^{2}/2E_{av}$ [52] (where *A* is the bearing cross-sectional area of the sample; *h* is the height of the sample; *E*_av_ is the average elastic modulus of the sample).

**Table 1:** The mechanical and ultrasonic testing results of coal and rock bodies around boreholes.

Sample number	Grouting materials	*σ*_p_(MPa)		*U*_e_(J)		*A*_p_(V)		*V*_p_(m/s)		*α*(dB·m^-1^)	
		Test value	Average value	Test value	Average value	Test value	Average value	Test value	Average value	Test value	Average value
**SH-BH-1**	-	8.24	8.36	22.83	23.43	555	536	2790	2610	49.28	49.26
**SH-BH-2**		8.51		24.35		522		2607		49.30	
**SH-BH-3**		8.33		23.11		531		2570		49.20	
**SH-PU-1**	Polyurethane	9.32	9.32	26.37	26.35	1194	1178	3040	3040	17.65	17.75
**SH-PU-2**		9.3 8		26.47		1153		2970		18.01	
**SH-PU-3**		9.26		26.03		1189		3010		17.59	
**SH-CEM-1**	Expansive cement	13.26	13.41	37.36	37.38	1433	1410	3450	3430	9.30	9.28
**SH-CEM-2**		13.59		37.42		1382		3360		9.27	
**SH-CEM-3**		13.38		37.36		1416		3427		9.27	

The strength of the grouting material affects the mechanical characteristics, energy dissipation characteristics, deformation failure process and ultrasonic propagation characteristics of the coal and rock bodies around boreholes. Compared with SH-BH, the peak stress of SH-PU increased by 0.96 MPa, the elastic energy at the peak increased by 12.46%, the first ultrasonic amplitude at the peak increased by 2.20 times, and the ultrasonic velocity increased by 16.48%. When expansion cement was injected into the borehole, the compressive strength of the coal and rock bodies around boreholes increases greatly. Compared with SH-BH, the peak stress of SH-CEM increased by 5.05 MPa, the elastic energy at the peak increased by 59.54%, the first ultrasonic amplitude at the peak increased by 2.63 times, and the ultrasonic velocity increased by 31.42%.

**Fig 4:**
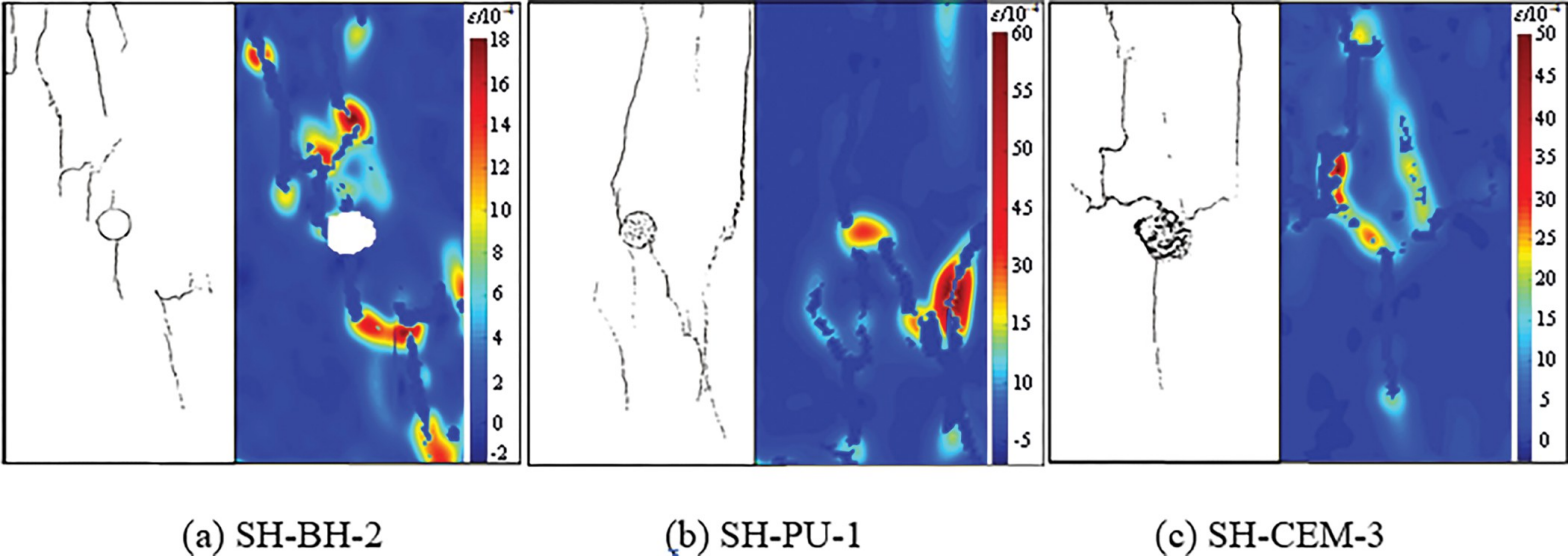
The sketches of the failure form and the cloud images of strain field.

According to the test results of DIC, the sketches of the failure form and the cloud images of strain field were drawn. The results are shown in Fig 4.

It can be found that the strength of grouting material has an important influence on the failure form and strain field distribution of the sample. Among them, two cracks through the whole sample were produced on the upper and lower sides of the borehole of SH-BH, and more cracks were developed at the end position of the sample. The failure of the sample was serious and the cracks were fully developed. Due to the relatively weak strength and cohesion of polyurethane, compared with SH-BH, SH-PU produced two cracks through the whole sample on both sides of the borehole, and the cracks were fully developed, but there were only a few cracks at the end of the sample. At the same time, there is obvious strain concentration at the borehole of SH-PU during failure, and the deformation of borehole is reduced compared with that of SH-BH. After injecting expansive cement into boreholes, the failure zone of SH-CEM is mainly concentrated around boreholes, only two main cracks extending to the end of the sample, and the crack width is small. In the case of expansive cement injection, the whole strain field distribution is relatively uniform, and there is no strain localization phenomenon due to the existence of prefabricated boreholes. The strain concentration at the boreholes was not obvious the failure.

## Results and discussion

### Ultrasonic waveform characteristics

**Fig 5:**
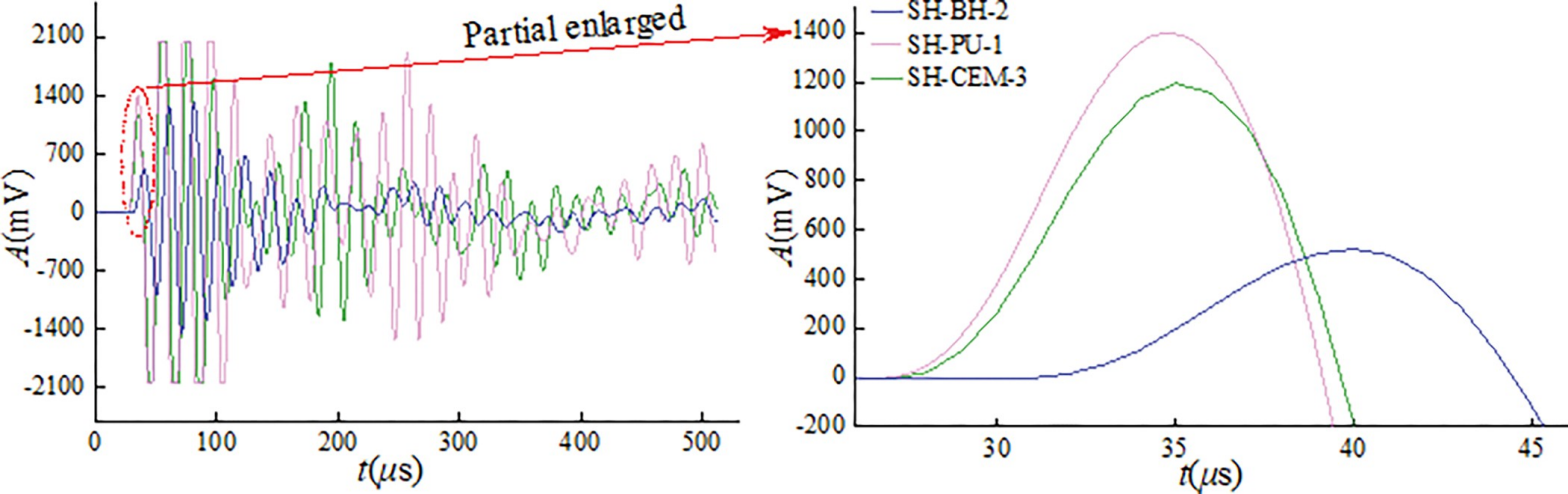
Initial ultrasonic amplitude of samples.

Fig 5 shows the initial ultrasonic amplitude of three groups of samples. In the time domain, the ultrasonic waveform shapes of the three groups of samples filled with different materials were basically the same, indicating that the ultrasonic was collected well. There is no failure inside samples in the initial state, which had little effect on the propagation of ultrasonic. When the ultrasonic passes through the sample, multiple reflections and diffractions will occur inside the sample. The vibration associated with ultrasonic wave transmission and the ultrasonic wave generated by the ultrasonic instrument will superimpose to form resonance, which is expressed as the significant increase in the amplitude of the second and third peaks.

Analysis of Fig 5 shows that the existence of grouting materials has an important influence on the transmission of ultrasonic waves. The first wave of SH-CEM-3 reached the peak at 1463mV, the ultrasonic energy decayed slowly in the time domain, and the coda wave continued to develop. Followed by SH-PU-1, with a peak at 1194mV. Finally for the SH-BH-2 wave peak of 522mV, and ultrasonic energy in the time domain decay fastest, the ultrasonic amplitude region smooths with the increase in time, and the coda wave does not develop. The arrival time of SH-BH-2 to the wave peak is delayed by 7μs compared to SH-CEM-3. This is because the existence of boreholes directly affects the transmission of ultrasonic waves, resulting in a longer time for the ultrasonic waves to reach the first wave amplitude, and a decrease in amplitude. When the ultrasonic wave transmitted in the grouted sample with borehole, the grouting material is more beneficial to the transmission of the acoustic wave. The transmission characteristics change less compared to non-grouted samples, and the first wave amplitude is higher.

**Fig 6:**
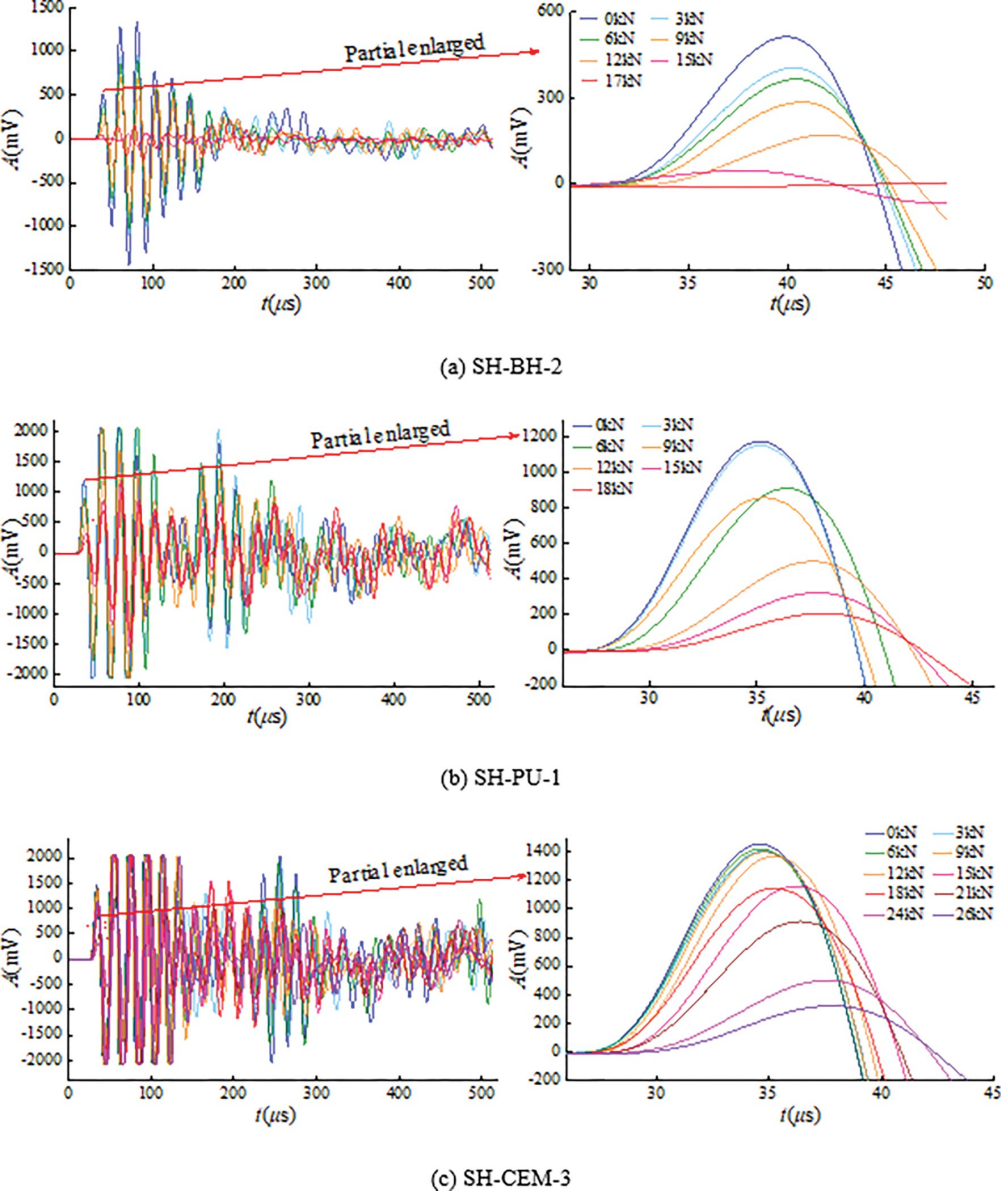
Ultrasonic waveform of coal and rock bodies around boreholes during progressive failure.

In order to analyze the change law of wave form of coal and rock bodies around boreholes during the progressive failure, the ultrasonic waveform data of three groups of samples during the loading are drawn, as shown in Fig 6. Among them, 0kN represents the waveform data of the ultrasonic wave through the sample before the test starts. 3~26kN corresponds to the waveform data collected by the ultrasonic instrument when the testing machine is loaded to 3~26kN, respectively. Because there are many ultrasonic wave data collected of each group of samples during the loading, the waveform data measured at every 3kN is selected for analysis in this paper, which can well explain the ultrasonic wave transmission process of the sample during the failure [53].

According to the analysis of Fig 6, the waveforms of SH-BH-2 and SH-PU-1 are relatively consistent when the stress is 0~9kN, and there is only a delay of 1~2μs in time. It shows that the failure degree of the sample in this stage is small. However, a large number of cracks inside the sample began to propagate after 9kN. The cracks were bypassed by ultrasonic many times during the ultrasonic transmission, which would cause partial delay (9μs) and eventually lead to a decrease in amplitude. While SH-CEM-3 has the highest waveform similarity throughout the loading process, the overall waveform shape is basically the same, and there is only a 3μs delay in time, indicating that the waveform collection is good and SH-CEM-3 is less destructive and has little effect on ultrasonic transmission. SH-CEM-3 has the highest waveform similarity in the whole loading process, the overall waveform shape is basically the same, and there is only 3μs delay in time. It shows that the waveform collection is good, SH-CEM-3 has less failure and has little effect on ultrasonic transmission.

The first ultrasonic amplitude decreases by 517mV for SH-BH-2, by 979mV for SH-PU-1 and by 1132mV for SH-CEM-3 during the whole loading. This reflects that in the case of borehole with grouting, the strength of the grouting material itself will make the sample start to crack only after a longer time. The higher the strength of the grouting material, the higher the compressive strength of the sample, the longer the time for crack propagation, and the slower the ultrasonic decay.

### Ultrasonic velocity characteristics

**Fig 7:**
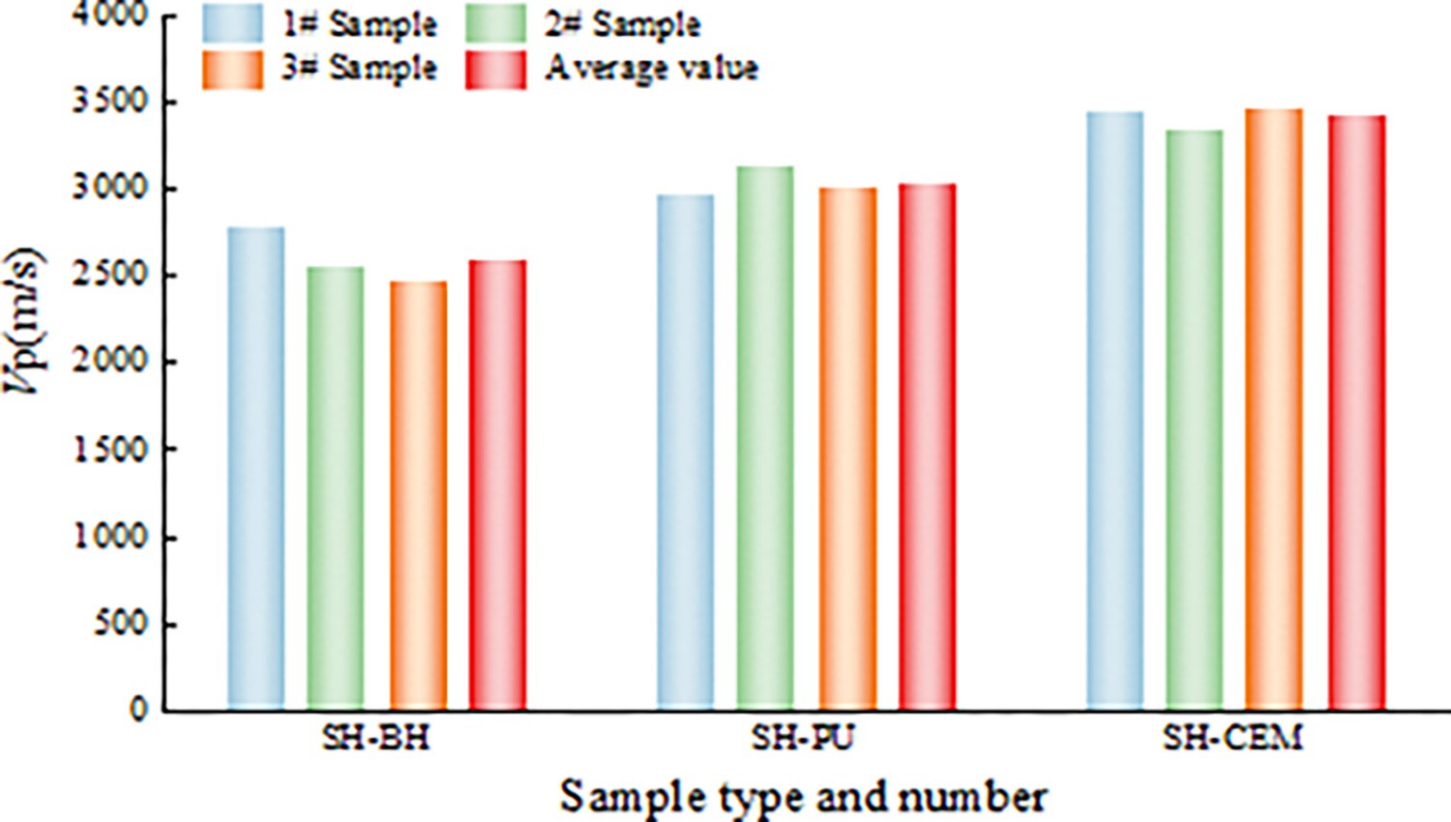
Initial ultrasonic velocity of samples.

According to the initial waveform data of the samples, the initial ultrasonic velocity of the three groups of samples is calculated, as shown in Fig 7. The initial ultrasonic velocity of the SH-BH group samples ranged from 2470 to 2790m/s with an average ultrasonic velocity of 2610 m/s. The initial ultrasonic velocity of the SH-PU group samples ranged from 2970 to 3140m/s with an average ultrasonic velocity of 3040m/s. The initial ultrasonic velocity of the SH-CEM group samples ranged from 3360 to 3480m/s with an average ultrasonic velocity of 3430m/s. Although there are small fluctuations in the initial ultrasonic velocity of the same type of samples, their overall mean values show a gradual increase in the initial ultrasonic velocity with the increase in the strength of the grouted material.

**Fig 8:**
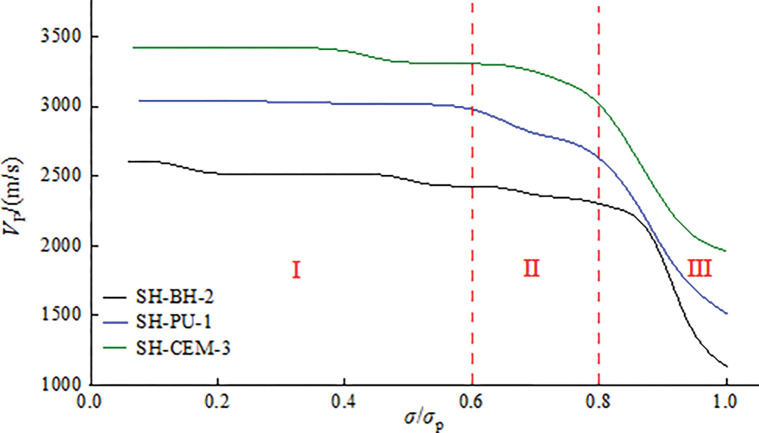
Ultrasonic velocity of coal and rock bodies around boreholes during progressive failure.

The stress level can reflect the propagation process of axial tension cracks during the deformation and failure of the sample, which can correspond to the transmission process of ultrasonic [54]. The sample failure process corresponds to three stress levels, so the relationship between the ultrasonic velocity and stress level of the coal and rock bodies around boreholes during progressive failure can be obtained, as shown in Fig 8.

It can be seen from Fig 8 that the overall change trend of the ultrasonic velocity of the samples is consistent during the loading, showing three stages of stable (I), slowly decrease (II) and rapidly decrease (III). This is consistent with the research results of the surface deformation of the coal and rock bodies around boreholes [55], and the ultrasonic velocity measurement results are also consistent with the ultrasonic velocity change type proposed by Hu et al [56].

In the region Ⅰ in Fig 8, the ultrasonic velocity remains basically constant at the early stage of loading (stress level 0<*σ*/*σ*_p_<0.6). The ultrasonic velocity of SH-BH-2 remains between 2570~2790m/s; the ultrasonic velocity of SH-PU-1 remains between 2970~3040m/s; the ultrasonic velocity of SH-CEM-3 remains between 3360~3450m/s. The variation of ultrasonic velocity is small, which indicated that the failure inside the sample is low at this time. In region II, the ultrasonic velocity of the samples decreases slowly in the middle of loading (stress level 0.6<*σ*/*σ*_p_<0.8), which indicates that internal failure and gradual formation of cracks in the sample. With the further increase of the loading stress, the sample enters the late stage of loading (region III, stress level 0.8<*σ*/*σ*_p_<1.0), the ultrasonic velocity is significantly reduced to the minimum. After the failure of coal and rock bodies under loading pressure, its internal space is filled with various macrocracks. These cracks propagate and connect, which makes the carrying capacity and integrity of coal and rock bodies weaken significantly. At this stage, the critical failure ultrasonic velocity of SH-BH-2 is 2270m/s, the critical failure ultrasonic velocity of SH-PU-1 is 2760m/s, the critical failure ultrasonic velocity of SH-CEM-3 is 3080m/s.

During the whole loading stage, the ultrasonic velocity of SH-BH-2 is decreased by 1780m/s; the ultrasonic velocity of SH-PU-1 is decreased by 1530m/s; the ultrasonic velocity of SH-CEM-3 is decreased by 1320m/s. Especially in stage III, the ultrasonic velocity decrease accounts for more than 50% of the total decrease. At this time, a large number of cracks inside the sample are connected, which leads to the final failure of the sample and causes ultrasonic attenuation. It can be obtained that when the sample is damaged, the crack propagation is the main reason for the decrease of ultrasonic velocity, and the change of ultrasonic velocity reflects the change of stress in the sample during the loading process. The change rate of the ultrasonic velocity is different for samples containing boreholes filled with different grouting materials.

### Ultrasonic attenuation characteristics

**Fig 9:**
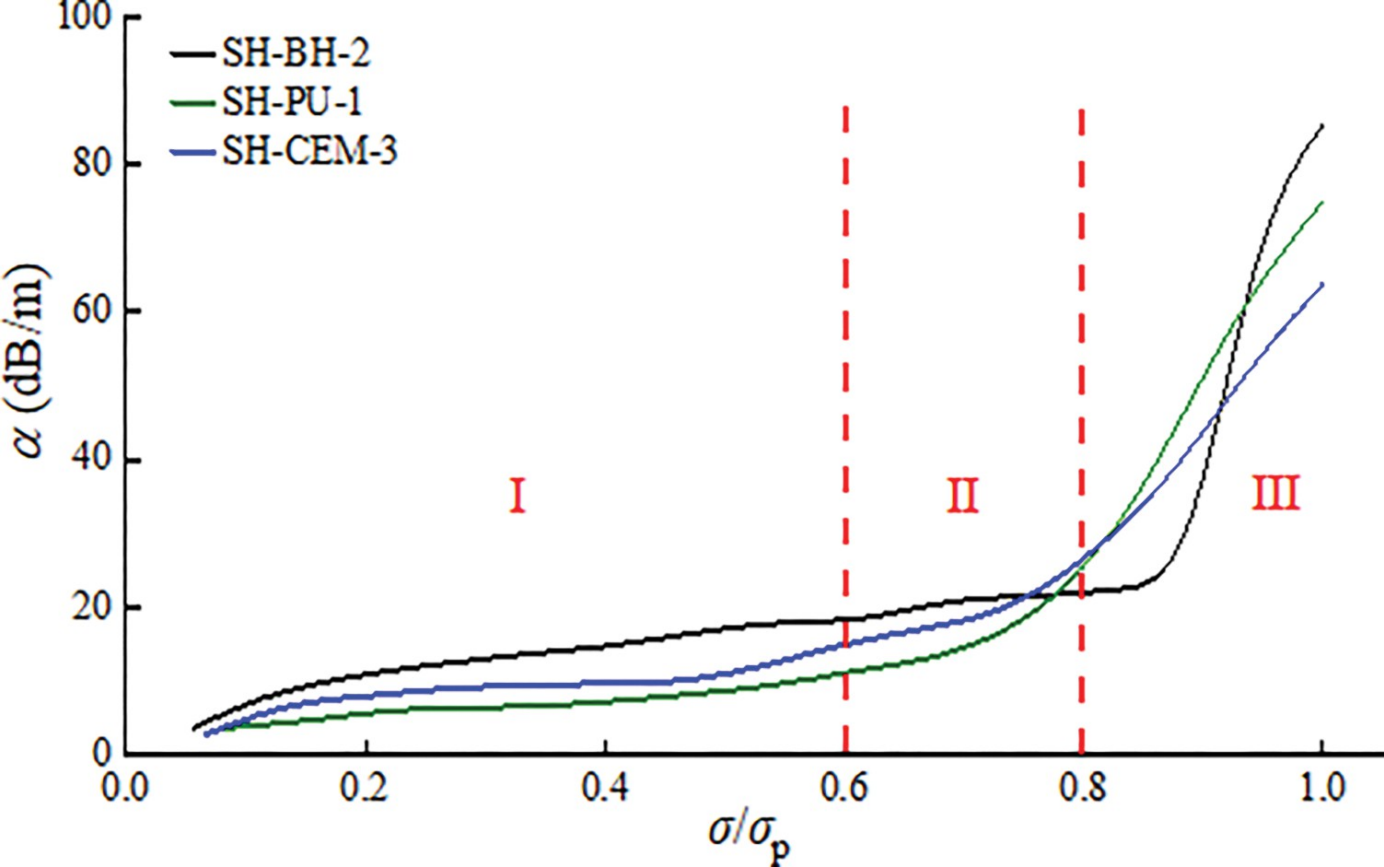
Ultrasonic attenuation of coal and rock bodies around boreholes during progressive failure.

The ultrasonic attenuation coefficient (*α*) reflects the degree of ultrasonic energy loss, which can reflect the degree of deformation failure and crack evolution in coal and rock bodies [57]. Therefore, the attenuation coefficients of the coal and rock bodies around borehole during progressive failure were analyzed to better understand the transmission process of ultrasonic waves inside the coal and rock bodies. The results of ultrasonic attenuation coefficient test for three groups of samples are shown in Fig 9.

It can be seen from Fig 9 that at the initial stage of loading, the ultrasonic attenuation coefficients of the three groups of samples changed more slowly, but the overall trend increased. It reflects that the direction of lateral deformation inside the sample at this stage has gradually appeared. After that, the attenuation coefficient shows an increasing trend, especially in the later stage of loading. The changes of the attenuation coefficients at the early, middle and late stages of loading reflect the internal deformation, failure and crack evolution of the sample, corresponding to the failure process of the coal and rock bodies. The attenuation coefficients show three stages of stability (I), slow increase (II) and rapid increase (III).

### Equivalent crack width

Figs 8 and 9 show almost the same response behavior between different ultrasonic parameters during progressive failure. When the loading stress is about 0.6*σ*_p_, the ultrasonic parameters begin to change gradually, indicating that the number of cracks in the sample begins to increase. In this paper, an equivalent crack width model based on ultrasonic velocity will be established to discuss this phenomenon in detail.

**Fig 10:**
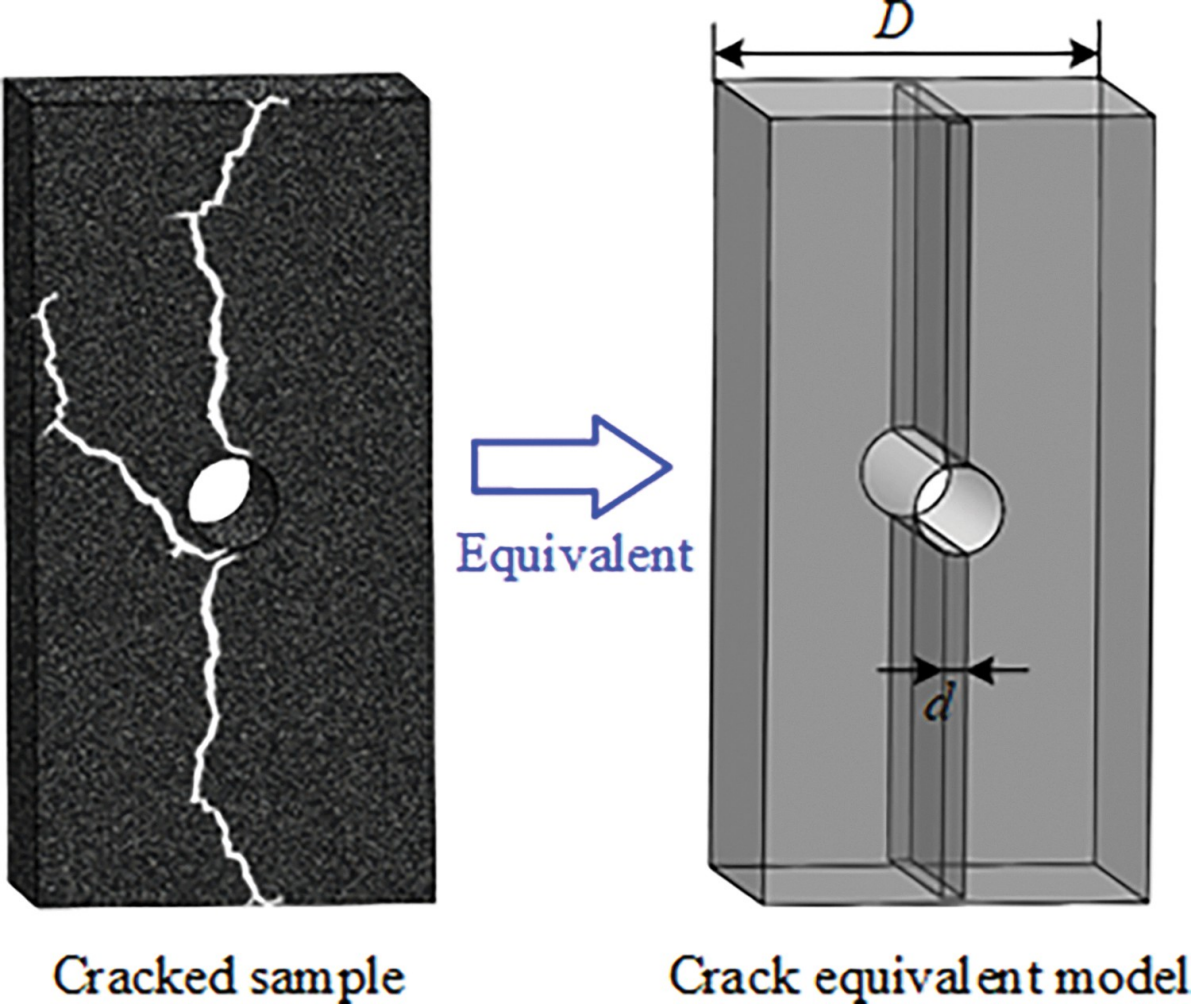
Equivalent crack width model of the sample.

Zhang et al [39] studied show that when cracks initiate and expand inside the sample, the cracks space will be filled with water or air. Since the propagation velocity of ultrasonic in solids is much faster than that in water or air, the ultrasonic velocity will decrease significantly when the number of cracks in the sample increase. In this paper, assuming that the width of the sample is *D*, the multiple irregular cracks in the sample during progressive failure are equivalent to air medium with a width of *d*_v_ in the middle of the sample, as shown in Fig 10.

Therefore, when the ultrasonic passes through the cracked coal and rock bodies, the relationship between equivalent crack width and ultrasonic velocity is as follows:

$$\frac{D-d_{v}}{V_{0}}+\frac{d_{v}}{V_{1}}=t$$ (3)

Where *V*_0_ is the velocity of ultrasonic propagation in non-cracking coal and rock bodies; *V*_1_ is the velocity of ultrasonic propagation in the air; *t* is the time when ultrasonic passes through cracked coal and rock bodies.

According to Eq (3), the equation of equivalent crack width *d*_v_ based on ultrasonic velocity is:

$$d_{v}=V_{1}\cdot (V_{0}t-D)/(V_{0}-V_{1})$$ (4)

According to the above equation, it is necessary to carry out ultrasonic waveform processing on the basis of ultrasonic test of coal and rock bodies around boreholes before and after grouting sealing. Since the first ultrasonic peak in the measured ultrasonic waveform reaches the left side of the sample through the shortest path, which can be approximated as a straight line. It is considered that there is no waveform superposition. Therefore, the first wave is very important for analysis, and the equivalent crack width can be calculated by the initial reach time of the first wave [58].

**Fig 11:**
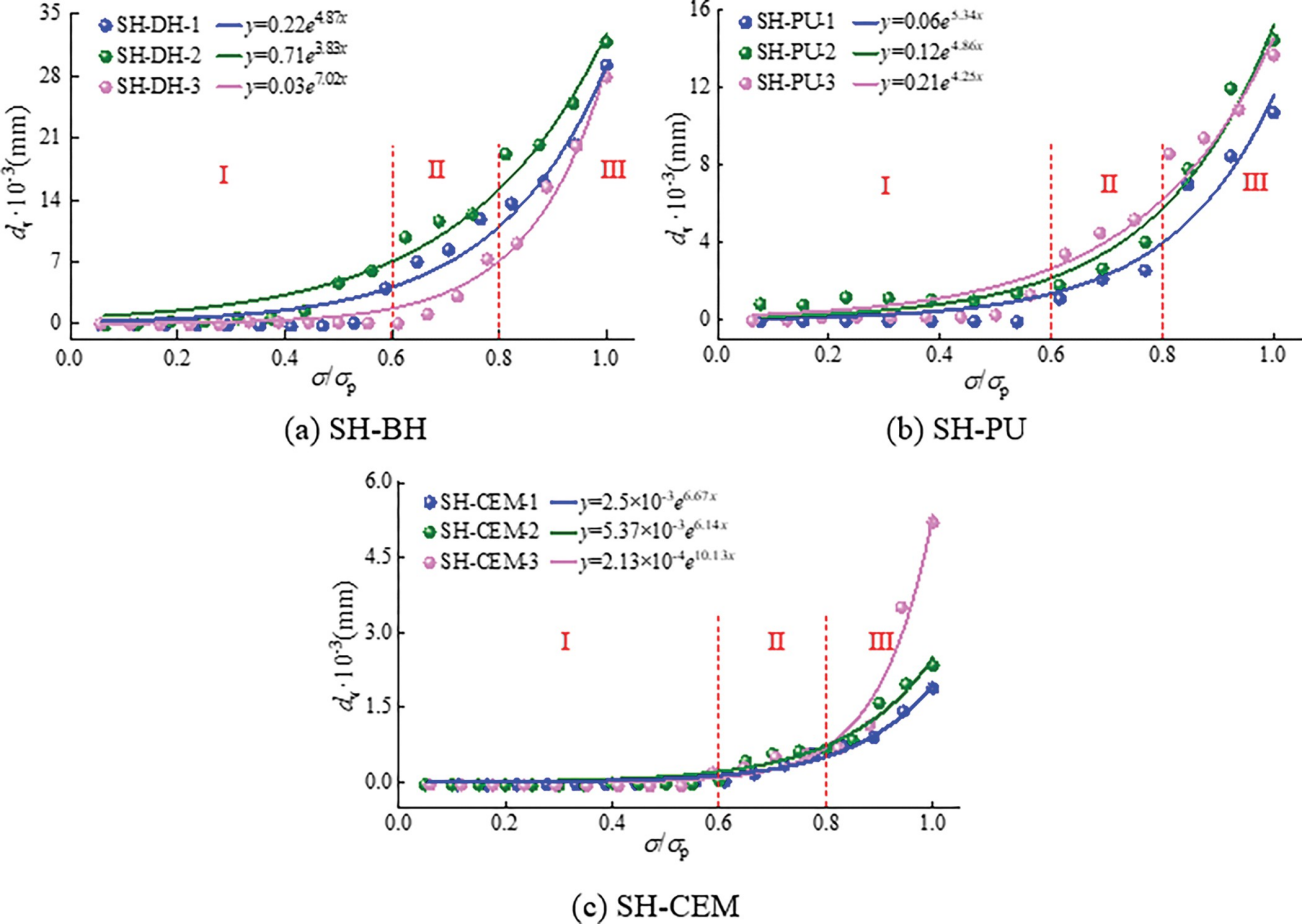
Equivalent crack width evolution curve of the sample during progressive failure.

The relationship between the equivalent crack width and stress level in the three groups of samples during loading was analyzed according to the equivalent crack width formula, as shown in Fig 11.

In Fig 11, the equivalent crack width of the sample and its stress level satisfiy the exponential relationship. With the increase of stress level, the equivalent crack width gradually increases. At the stress level is 0.6*σ*_p_, the equivalent crack width begins to increase slowly, and then increases rapidly at 0.8*σ*_p_, which corresponds to the response characteristics of ultrasonic parameters during progressive failure. By fitting, the relationship between equivalent crack width and stress level of coal and rock bodies around boreholes before and after grouting sealing is obtained.

$$d_{v}=ae^{b(\sigma /\sigma_{p})}$$ (5)

Analysis of Fig 11 shows that at the initial stage of loading, when the stress level is 0<*σ*/*σ*_p_<0.6, the equivalent crack width remained near 0. The sample is in the initial stage of failure, the internal failure degree of the sample is low, and only a small amount of micro-cracks are produced. The equivalent crack width of the sample increases gradually when the stress level is 0.6<*σ*/*σ*_p_<0.8, indicating that the internal cracks begin to propagation in the middle stage of loading. At the later stage of loading, when the stress level is *σ*/*σ*_p_>0.8, the equivalent crack width increases rapidly, indicating that the sample begins to accelerate the failure and the internal crack propagation forms macroscopic cracks. The propagation path of ultrasonic becomes from the solid medium of the sample into the crack, through the crack and into the solid medium again, and finally received by the receiver of the ultrasonic transducer.

At the same time, the strength of the grouting material seriously affects the equivalent crack width, and the equivalent crack width of the sample decreases with the increase of the strength of the grouting material. As the equivalent crack width model of sample shows: in this process, due to the ultrasonic velocity in the air is 340m/s, which is much smaller than the ultrasonic velocity of the coal and rock bodies in the initial state, resulting in the first ultrasonic arrival time delay. Finally, at the time of reaching the peak stress, the equivalent crack width of SH-BH increases about 0.027mm~0.032mm, SH-PU about 0.01mm~0.014mm, and SH-CEM about 0.002mm~0.006mm.

In this paper, ultrasonic technology was used to study the crack propagation law of coal and rock bodies around boreholes during progressive failure, and a series of valuable conclusions are obtained. Using the ultrasonic testing results of coal and rock bodies around boreholes, the crack propagation around the gas extraction boreholes is evaluated, which can provide a theoretical basis for the application of ultrasonic testing in gas extraction borehole leakage. However, due to the special characteristics of coal and rock bodies around boreholes and the limitation of laboratory conditions, the ultrasonic parameters of ultrasonic signals and the quantitative analysis of internal cracks in coal and rock bodies around boreholes during progressive failure need to be further studied. In addition, the following work can be carried out in the follow-up study: (1) the ultrasonic characteristics of the specimen during the whole loading process, as well as the response of ultrasonic amplitude, ultrasonic velocity and ultrasonic attenuation coefficient to the total stress level were analyzed. (2) Based on elastic wave theory, fracturing mechanics and damage mechanics, SEM and CT scanning tests were carried out to reveal the relationship between crack propagation characteristics, crack width and ultrasonic parameters.

## Conclusion

In this paper, the ultrasonic testing system for deformation and failure of coal and rock bodies is used to carry out the ultrasonic testing test of coal and rock bodies around boreholes under uniaxial compression. The mechanical properties, deformation and failure process, ultrasonic propagation characteristics and crack propagation law of samples containing different grouting boreholes are analysed. The following conclusions are drawn:

(1) The strength of the grouting material significantly affects the mechanical characteristics, energy dissipation characteristics, deformation and failure processes and ultrasonic propagation characteristics of the coal and rock bodies around borehole. As the strength of the grouting material increases, the peak strength of the sample *σ*_p_, the elastic energy at the peak *U*_e_, the first ultrasonic amplitude *A* and the ultrasonic velocity *V*_p_ all gradually increase, while the surface displacement of the samples and the ultrasonic attenuation coefficient *α* decrease gradually. Finally, the surface cracks on the samples show tensile failure, scattered on both sides of boreholes, expanding and slipping along the left and right sides of boreholes, forming shear diagonal cracks.

(2) During the loading process, the arrival time of the first wave of the SH-BH-2 is 8μs, the ultrasonic energy decays fastest in the time domain, the amplitude of the ultrasonic gradually stabilizes with time, and the coda wave is not developed. But the failure degree of SH-CEM-3 is small, the waveform similarity is the highest, the overall waveform shape is basically the same, only 3 μs delay in time. The ultrasonic energy attenuates slowly, and the coda wave continues to develop.

(3) During the progressive failure, the ultrasonic velocity and attenuation coefficient all show three stages of stability(0~0.6*σ*_p_), slow change(0.6*σ*_p_~0.8*σ*_p_) and rapid change(0.8*σ*_p_~1.0*σ*_p_). Influenced by the strength of the grouting material, the three stages experienced different lengths of time. In the stage of rapid change of ultrasonic characteristic parameters, obvious cracks were produced on the surface of the sample. The "sudden decrease" of ultrasonic velocity and the "sudden increase" of ultrasonic attenuation coefficient can be used as the basis for judging the crack propagation of the coal and rock around borehole.

(3) The stress level reflects the crack expansion during the failure of the sample, and the failure process corresponds to 3 stress levels. The equivalent crack width increases exponentially with the increase of stress level during the failure of the sample, corresponding to the response characteristics of the ultrasonic parameters during progressive failure. The equivalent crack width decreases significantly with the increase of the strength of the grouting material. At the time of reaching the peak stress, the equivalent crack width of SH-BH grows about 0.027mm~0.032mm, SH-PU about 0.01mm~0.014mm, and SH-CEM about 0.002mm~0.006mm.

## Supporting information

S1 Data (RAR)
